# Ferreting Out the Effects of Neonatal Hypoxia–Ischemia and Sex on Ferret Cortical Gyrification

**DOI:** 10.3390/life15091428

**Published:** 2025-09-11

**Authors:** Olivia C. Brandon, Olivia R. White, Kylie A. Corry, Andreea Stanescu, Arian Ariaye, Daniel H. Moralejo, Janessa B. Law, Sarah E. Kolnik, Sandra E. Juul, Thomas R. Wood

**Affiliations:** 1 Division of Neonatology, University of Washington, Seattle, WA 98105, USA; obrandon@uw.edu (O.C.B.); arianthemg@gmail.com (A.A.); janessal@uw.edu (J.B.L.); sjuul@uw.edu (S.E.J.); 2 Institute on Human Development and Disability, University of Washington, Seattle, WA 98195, USA

**Keywords:** gyrification, hypoxia–ischemia, neonatal, erythropoietin, therapeutic hypothermia

## Abstract

Gyrification, the folding of the cerebral cortex, plays a crucial role in brain development and function. Perinatal hypoxia–ischemia (HI) is a leading cause of neonatal brain injury, affecting cortical folding that can be measured by the gyrification index (GI). Using a late-preterm ferret model, our objective was to explore the relationships between HI injury, GI changes, and behavior, as well as the potential moderating effects of sex and treatment. Animals received 3 mg/kg *E. coli* lipopolysaccharide and underwent bilateral carotid artery ligation followed by alternating hypoxia and hyperoxia (HIH) and were randomized to saline vehicle (*n* = 25), erythropoietin (*n* = 20), therapeutic hypothermia (6 h at 33.5 °C, *n* = 20), and uridine monophosphate (*n* = 6), with *n* = 20 unexposed littermates serving as controls. Early reflex testing, CatWalk gait analysis, open-field behavior, and an open-water swim test were performed. Average, peak, motor, and somatosensory strip GIs were then assessed using ex vivo MRI. In control animals, males had lower GI than females; however, HIH exposure resulted in male GI being more similar to females, where HIH animals had significantly higher average GI than controls (*p* = 0.02). Adjusting for brain volume and injury, GIs in motor and somatosensory areas were associated with faster reflex outcomes in males but not females. In female controls, motor and somatosensory GIs were associated with increased anxiety-like behaviors, such as spending less time in open water during the swim test. By comparison, in male controls, higher GI was associated with decreased anxiety-like behaviors, including higher exploration index in the swim test. These sex-specific relationships between GI and behavior were lost with HIH injury. Treatment did not meaningfully restore the relationship between GI and behavior after HIH, but targeting this outcome may be an important measure for use in future neuroprotection studies in the ferret.

## 1. Introduction

Perinatal hypoxia–ischemia (HI) is a leading cause of neonatal morbidity and mortality [1,2]. The current standard of care for term newborns with HI is therapeutic hypothermia (TH), but HI also frequently occurs in late-preterm infants for whom TH may not be neuroprotective [3]. TH is also only available in high-resource settings and only provides partial protection—at least 30% of infants who receive TH still experience death or disability [4,5]. In addition, TH may not be neuroprotective in lower-resource settings and even increases adverse outcomes [6]. Therefore, the search for additional neuroprotective treatments for HI is critical.

The ferret (*Mustela putorius furo*) provides a promising model to improve translation of therapies for HI due to its mid-sized brain, white-to-gray matter ratio that is more similar to humans compared to rodents, and a gyrified cerebral cortex [7,8,9,10,11,12]. In our ferret model of late-preterm inflammation-sensitized HI, erythropoietin (Epo), but not TH, reduces neuropathological injury [7].

As we continue the search for additional neuroprotective agents for infants with HI, especially those born preterm, reliable tools to assess normal and pathological brain development become increasingly important. Rapid cortical folding occurs during the late second and third trimesters, with much of this process taking place before term birth [13]. In preterm infants, disruptions to this critical period of gyrification have been linked to neurodevelopmental impairments, making cortical folding a relevant marker for brain maturation and later outcomes [14].

The gyrification index (GI) has been proposed as one such tool to assess cortical development in gyrified species such as humans and ferrets, with known sex differences in gyrification over time [15]. For example, a study by Kline et al. (2020) found that GI was associated with neurodevelopmental outcomes in preterm infants [16]. GI in ferrets is altered by neurotoxic exposures, including decreased GI in ferrets exposed to valproic acid during development [17]. However, the effect of experimentally induced HI, as well as subsequent treatment with therapies such as Epo and TH, on GI is unknown.

We sought to evaluate the effects of sex and treatment on post-HI gyrification in a late-preterm HI ferret model. We hypothesized that (1) GI would be affected by injury and that this effect would differ by sex, (2) GI in the motor strip of the cortex would be associated with behavioral tests involving gait development and motor function, (3) GI in the somatosensory strip would be associated with reflex development given its critical role in integrating sensory information, and (4) treatments would mitigate or accentuate GI-associated behavioral outcomes.

## 2. Materials and Methods

### 2.1. Animals and Housing

Experiments were performed in strict accordance with the recommendations of the National Institutes of Health Guide for the Care and Use of Laboratory Animals, ARRIVE guidelines (Animal Research: Reporting of In Vivo Experiments), and were approved by the University of Washington Institutional Animal Care and Use Committee (IACUC, protocol #3328-06 and #3328-07). Sex-balanced litters of ferrets (*n* = 4 per sex) were ordered from Marshall Bioresources (North Rose, NY, USA) and arrived on postnatal (P) day 8. Full methods and formal neuropathological outcomes from these animals have previously been published [7].

### 2.2. Hypoxic–Ischemic Injury Model

On P17, which is 32–36 weeks human gestational age equivalent, kits were jill-separated, weighed, and randomly assigned to either control or inflammation pre-sensitized hypoxia–ischemia with hyperoxia (HIH) [9,11]. Animals assigned to the HIH group received 3 mg/kg i.p. lipopolysaccharide (LPS; Ultrapure from *E. coli* 055:B5, Lot #4231A, List Biological, Campbell, CA, USA). LPS was given approximately 4 h before hypoxia to synchronize timing of hypoxia exposure with the peak inflammatory response [18,19].

All HIH animals underwent bilateral carotid artery ligation under isoflurane anesthesia (3–5%) and buprenorphine (0.05 mg/kg) analgesia. The left carotid artery (LCA) was permanently ligated using silk suture (5–0, Fine Science Tools, Foster City, CA, USA), and the right carotid artery (RCA) was temporarily occluded with umbilical tape (1/8-inch, GF Health Products, Atlanta, Georgia, USA) [9]. After a 30 min recovery, all ferret kits that underwent surgery were then exposed to hypoxia–hyperoxia–hypoxia (9%, 80%, and 9% oxygen each for 30 min). Following the last hypoxia exposure, the RCA occlusion was removed.

After HIH, kits were returned to their home cages with the jills for a 1 h recovery period. HIH-exposed animals were then randomized to saline vehicle (vehicle), Epo (2000 IU/kg delivered subcutaneously at 0 h, 24 h, 48 h, and 7 days after HIH), TH (33.5 degrees Celsius for 6 h), or uridine 5′ monophosphate (Sigma cat. U1752, St. Louis, MO, USA; delivered subcutaneously at a concentration of 500 mg/kg every 12 h for 5 doses). All treatments were initiated within 90 to 120 min after the HIH exposure. Animals assigned to TH were kept in a separate water bath adjusted to keep target rectal temperature at 33.5 degrees Celsius while vehicle, Epo, and uridine animals were removed from the nest for a six-hour temperature-controlled normothermia period. A probe was used to measure rectal temperatures to adjust water baths to maintain animal temperature at either 33.5 or 37 degrees Celsius (Precision 4000A thermometer, YSI, Yellow Spring, OH, USA), respectively. Kits were then returned to their nests.

### 2.3. Behavioral Testing

Early reflex testing was conducted daily from P21 to P27 and three times per week from P28 to P39. Reflex testing included negative geotaxis at 25 and 45 degrees, cliff aversion, and righting reflex. Three trials for negative geotaxis and cliff aversion and five trials for righting reflex were collected. The area under the curve (AUC) across all tests conducted P21 to P39 was calculated, as previously published [7].

Animals had late behavioral testing that included CatWalk gait analysis (CatWalk XT, Noldus, Leesburg, VA, USA) on P42, early-childhood equivalent, as previously published [7]. Animals were required to complete 3 compliant runs, which included completing the runway of the CatWalk in under 10 s with a maximum speed variation of 60%. Numerous variables were analyzed to evaluate gait development with the CatWalk technology including paw area, base of support, and gait velocity. For open field, animals were placed into a 55 cm by 55 cm by 63.5 cm acrylic box with opaque walls on P42, and their behavior was monitored for 5 min using EthoVisionXT software (Stoelting Co., Wood Dale, IL, USA). Variables included total distance moved, velocity, time spent moving, and time spent in the zone near littermates (an 18 cm by 18 cm area adjacent to one side of the arena where littermates were nearby but remained outside the open field). The open-water swim test was conducted on P42 and included placing the animals into a pool of water for 60 s trials, including a 60 s acclimation period prior to data collection. EthoVision software (version 12, Noldus Information Technology, Wageningen, The Netherlands) tracked the animals’ movement, and variables relating to velocity, distance travelled, and exploration were reported. An exploration index was calculated as the track per tub area multiplied by the total distance, divided by the swim time.

### 2.4. Gyrification Index

**Figure 1:**
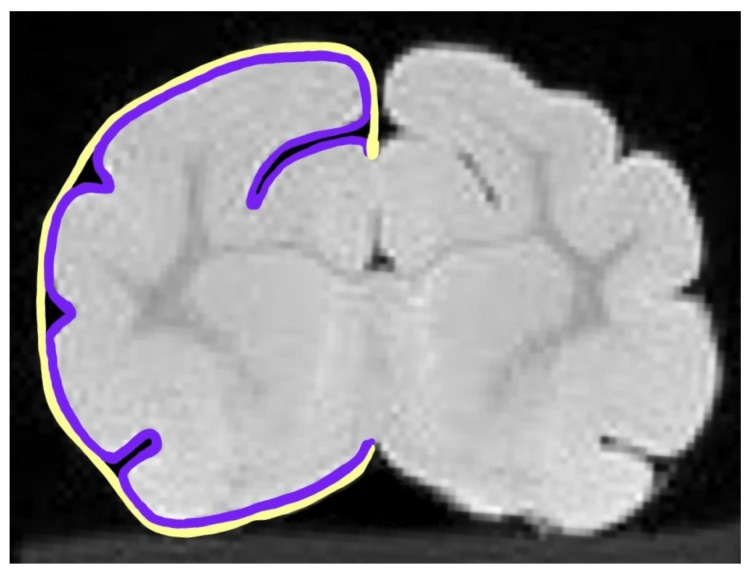
Example of inner (purple) and outer (yellow) tracings used to determine the gyrification index (GI) for each slice. The GI was calculated by dividing the inner trace that followed the curvature of the gyri and sulci by the outer trace that captured the cerebral circumference for each hemisphere.

Animals were euthanized on P42 and perfusion-fixed with phosphate-buffered saline (PBS) followed by 10% buffered formalin. Brains were stored in formalin for ex vivo magnetic resonance imaging (MRI). Brains were loaded onto agarose gel sleds within 50 mL Falcon tubes and immersed in Fomblin (Perfluoropolyether, PFPE; Solvay Specialty Polymers, Alpharetta, GA, USA). MRIs had a slice thickness of 0.23 mm and a sagittal slice orientation. Images were diffusion-weighted and collected with the Bruker Advance III, 4-7-tesla (200 MHz, 1 H), 20 cm horizontal-bore magnet with Para Vision (version 6.0.1). Brain volumes were quantified from MRI scans using automated segmentation and volumetric analysis. Based on previously published methodology, we calculated GI using MRIs of the cerebral cortex [15]. Using ImageJ software (version 1.53u) [20], each hemisphere was traced twice. The inner trace followed the contours of the gyri and sulci, and the outer trace followed the circumference of the cortex (Figure 1).

GI was calculated by taking a ratio of the inner trace to the outer trace. MRI images were traced across each slice of the entire brain starting immediately caudal to the olfactory bulbs until five sequential slices had a GI of less than 1.05, occurring around the disappearance of the posterior ectosylvian gyrus. For each slice, GI was determined by taking the average of the left and right hemisphere. Average GI was calculated across all traced slices per brain. Peak GI was determined by the highest average of the left and right hemispheric GI. The GI of the motor strip of the cortex was approximated to start with the presence of the cruciate sulcus and end with the disappearance of the anterior sigmoid gyrus, primarily evaluated by the angle of the splenic sulcus. The somatosensory cortex was approximated to start immediately behind the motor cortex and ended with the disappearance of the suprasylvian sulcus [21].

After MRI, coronal slices including the caudate nucleus were embedded in paraffin and underwent hematoxylin and eosin (H&E) staining. A board-certified veterinary pathologist evaluated the slides and created an overall pathology score of 0–20 including cortical lesion (scored 0–4), mineralization (0–4), sulci damage (0–4), gyri damage (0–4), and white matter thickness (0–4). More detailed MRI and pathology methodology have been previously published [7,8].

### 2.5. Statistical Analysis

Smoothed quantile regressions were used to display the median GI throughout the brain, split by hemisphere, sex, and treatments separately. When comparing HIH-exposed and control animals separated by sex, linear regressions with robust standard errors were used in R with the rigr package adjusted for brain volume modeled as a quadratic term to account for potential nonlinearity [22]. To evaluate the relationships between GI (average, peak, motor strip, and somatosensory strip), pathology score, and brain volume separated by sex and HIH exposure, both linear and quadratic models were analyzed and the model with the lowest Akaike information criterion (AIC) was selected. A reduction of at least two points in AIC was considered significantly lower, implying a better balance of fit and complexity. Due to the relationships between brain volume, pathology, and GI, we adjusted for brain volume and pathology in all analyses exploring the relationship between GI, behavior, and treatment. Brain volume was included as a quadratic term to account for potential nonlinearity.

While best statistical practice involves assessing interactions between sex and treatment [23], this study was not powered to do so after accounting for multiple treatment groups and the variability in injury. Due to the observable sex differences at the beginning of our analyses, we instead stratified animals by sex and performed analyses with males and females separately.

The relationships between motor and somatosensory strip GI and behavioral outcomes were explored to specifically assess relationships between the gyrification of distinct brain subregions and related behavioral testing outcomes. All models included adjustment for injury (HIH-exposed versus control), brain volume, pathology score, and the interaction between GI and HIH exposure. Linear regressions with robust standard errors were used, and scatter plots show the predicted marginal linear estimate with 95% confidence intervals from the final models. This method was also used to assess the effect of treatment stratified by sex. Uridine was excluded from treatment analyses due to small samples sizes (*n* = 2 female and *n* = 4 males) that resulted from increased mortality with treatment, as previously described [7].

As HIH altered GI, sensitivity analyses were performed restricting GI values to the ranges within control female and male animals to ensure that animals with GI values outside the range of controls did not drive any of the noted associations. This approach was applied for somatosensory strip and motor strip GI in female and male animals separately.

A probability (*p*) value less than 0.05 was considered significant for all analyses except for interactions in adjusted models, where *p* < 0.10 was considered significant due to the power required to detect significant interactions as well as the exploratory nature of the study that includes some willingness to accept an increase in type I error. All statistical analyses were performed in R Version 4.2.1 (Vienna, Austria) [24]. Additional figures were made in GraphPad Prism version 10.1.1 (GraphPad software, San Diego, CA, USA).

## 3. Results

### 3.1. GI Trends Across the Cortex and Hemispheres

**Figure 2:**
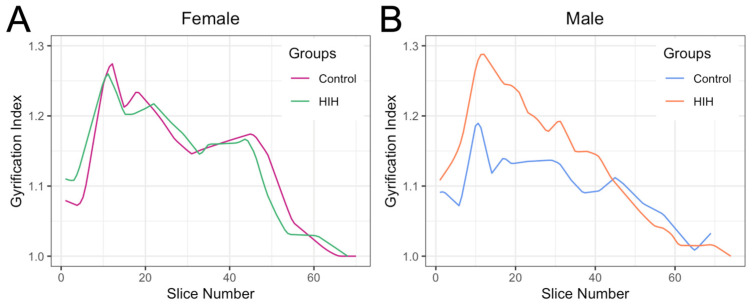
Smoothed quantile regression showing median gyrification index (GI) spanning the brain comparing control and HIH females (**A**) and males (**B**).

A total of 91 brains were traced for GI (*n* = 20 control, *n* = 25 vehicle, *n* = 20 Epo, *n* = 20 TH, and *n* = 6 uridine). The median (interquartile range; IQR) for number of images traced per brain was 59 (55, 64). Peak GI occurred approximately at slice 12 in both males and females (Figure 2), typically corresponding to where the cruciate sulcus reached its greatest depth and the posterior sigmoid gyrus was most prominent. Across all animals, average GI was 1.16 (1.13, 1.19), and peak GI was 1.39 (1.32, 1.43). Control animals showed similar patterns of GI across both hemispheres (Supplemental Figure S1A,B). Median GI in HIH-exposed animals appeared to show a higher peak on the left and more rostral peak on the right (Supplemental Figure S1A,B). However, no significant differences were found in average or peak GI in the left and right hemispheres stratified by sex (Supplemental Figure S1C,D); therefore, subsequent analyses show the GI averaged across both hemispheres for every slice. Due to HIH appearing to significantly alter global GI metrics only in males, all subsequent analyses were stratified by sex.

### 3.2. GI and Association with Injury Metrics

**Figure 3:**
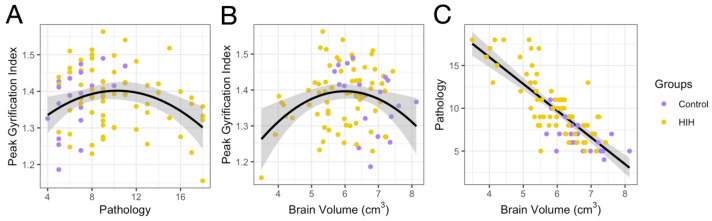
Quadratic regressions showing the association of peak gyrification index (GI) with brain pathology (**A**), brain volume (**B**), and a linear regression showing the association of brain volume and pathology (**C**) in control (purple) and HIH-exposed animals (yellow); 95% confidence interval shaded.

A quadratic model best fit the relationship between pathology and peak GI (Figure 3A). Peak GI also tended to increase as pathology increased, which we hypothesized might occur as the sulci deepened with injury, but then decreased again due to loss or complete lack of development of certain gyri with the most severe injury (Figure 3B). A linear model best fit the relationship between brain volume and pathology score: brain volume decreased significantly with increasing pathology score (Figure 3C). This relationship was also seen when control and HIH animals were examined separately (Supplemental Figure S2). A quadratic model also best fit the relationship between pathology score and average GI in both males and females (Supplemental Figure S3). Similar relationships were seen with motor and somatosensory GI with pathology and brain volume (Supplemental Figure S4). Due to pathology and brain volume being non-linearly associated with GI and injury, quadratic terms for these values were included as covariates in all subsequent models.

### 3.3. Effect of HIH on GI, Including Effect of Sex

**Figure 4:**
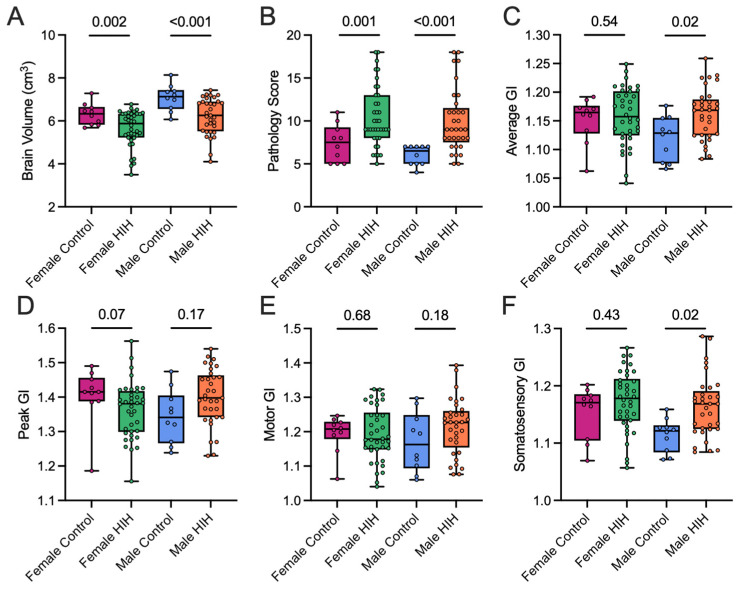
Box plots displaying brain volume (**A**), pathology score (**B**), average gyrification index (GI) (**C**), peak GI (**D**), motor GI (**E**), and somatosensory GI (**F**) grouped by sex and HIH-exposure. Both female and male HIH-exposed animals had significantly lower brain volumes (*p* = 0.002, *p* < 0.001, respectively) and higher pathology scores (*p* = 0.001, *p* < 0.001, respectively) compared to controls. Female control animals were similar to female HIH animals in both average and peak GI (*p* = 0.54, *p* = 0.07, respectively). Male HIH animals had significantly higher average GI compared to male control animals (*p* = 0.03) but had similar peak GI (*p* = 0.17). No significant differences were seen in motor GI. Male HIH animals had significantly higher somatosensory GI than controls (*p* = 0.02) while no difference was observed in females (*p* = 0.43). Linear regression with robust standard errors was used and all GI associations (panels (**C**–**F**)) were adjusted for brain volume modeled as a quadratic term to account for potential nonlinearity. Data are presented as a box plot, showing the median, interquartile range, and overall range.

HIH exposure also resulted in significantly decreased brain volumes (*p* = 0.002, *p* < 0.001) and increased pathology scores (*p* = 0.001 and *p* < 0.001; Figure 4A,B) in both females and males. Female control animals had an average GI of 1.17 (IQR: 1.14, 1.17) and a peak GI of 1.42 (1.39, 1.46). Similar to female controls, female HIH-exposed animals had an average GI of 1.16 (1.13, 1.20) and a peak GI of 1.38 (1.30, 1.41), neither of which was significantly different (*p* = 0.54, *p* = 0.07, respectively; Figure 4C,D). Male controls had an average GI of 1.13 (1.08, 1.15) and a peak GI of 1.34 (1.28, 1.39). After adjusting for brain volume, male HIH-exposed animals had significantly higher average GI of 1.17 (1.14, 1.18) but similar peak GI of 1.41 (1.38, 1.46) (*p* = 0.02, *p* = 0.17, respectively; Figure 4C,D) compared to male control animals.

There were no significant differences in motor GI with HIH exposure in females or males (*p* = 0.68 and *p* = 0.18, respectively, Figure 4E). Male controls animals had significantly lower somatosensory GI than male HIH-exposed animals (*p* = 0.02), but no effect of HIH was found in female animals (*p* = 0.43; Figure 4F).

**Figure 5:**
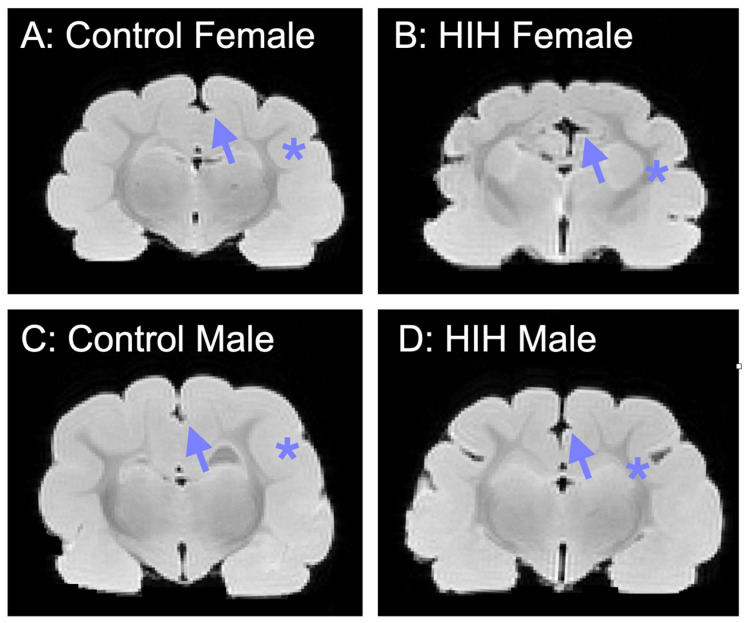
Example MRI images in control female (**A**), HIH female (**B**), control male (**C**), and HIH male (**D**) animals. In HIH males (**D**), brains show a deeper splenic sulcus (arrow), more similar to female animals (**A**,**B**). Other sulci at this level, such as the suprasylvian sulcus (*), were shallower in the control male animals compared to HIH males and females. Images from the median average GI for each sex and HIH exposure shown.

Representative images show that HIH-exposed males had a deeper splenic sulcus that was more similar to female animals (Figure 5). Control male animals also had shallower sulci, such as the suprasylvian sulcus, compared to HIH-exposed males and females.

### 3.4. Early Reflex Testing and GI

**Figure 6:**
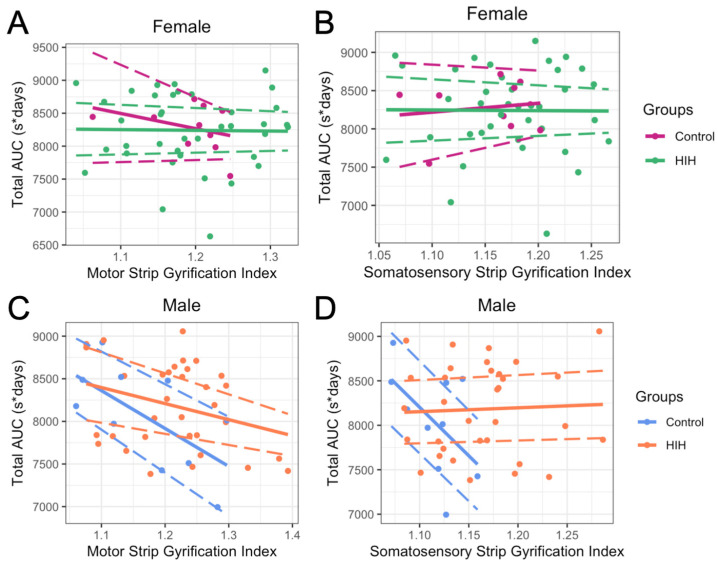
Relationship in females (**A**,**B**) and males (**C**,**D**) between motor strip gyrification index (GI) and somatosensory strip GI and total area under the curve (AUC) of reflex testing. Points represent individual data, and solid lines represent the predicted adjusted relationship from the linear model. Dashed lines indicate the 95% confidence intervals for the predictions. The model included an interaction between GI and HIH exposure, with brain volume and pathology as covariates. In females, motor and somatosensory GIs were not associated with reflex testing AUC after adjustment. In males, higher motor (*p* = 0.04) and somatosensory (*p* = 0.04) GIs were associated with faster reflex responses. This relationship was disrupted by HIH for somatosensory GI (interaction *p* = 0.06) but remained for motor GI (interaction *p* = 0.26). Asterisk (*) represents multiplication of seconds for task success and days to generate the AUC.

Motor and somatosensory GIs were associated with several behavioral outcomes (Supplemental Figure S5). After adjusting for HIH exposure, brain volume, pathology score, and the interaction between GI and HIH exposure, the main effect of GI, which represents the relationship in control animals, was not associated with reflex testing AUC in female animals (Figure 6A,B). However, the main effects of motor strip (*p* = 0.04) and somatosensory strip (*p* = 0.04) GIs were significantly associated with total reflex testing AUC (reflex performance over time, where a lower AUC represents faster reflex development) in male animals, with higher GI in control males associated with faster reflex development (Figure 6C,D). However, the association between somatosensory strip GI and reflex AUC seen in control males was lost with HIH exposure as the interaction between GI and HIH exposure was significant (*p* = 0.06, Figure 6D). In sensitivity analyses where GI metrics were restricted to the range observed in control animals, these sex-specific relationships by HIH exposure remained largely unchanged (Supplemental Figure S6).

### 3.5. Late Behavioral Testing and GI

**Figure 7:**
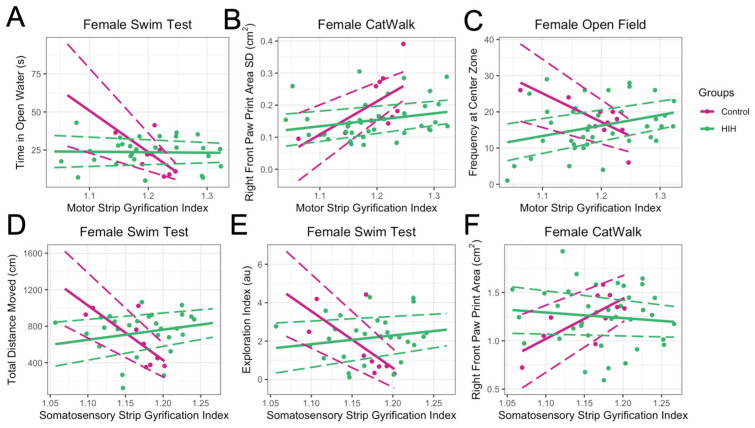
Relationship between gyrification index (GI) and behavioral outcomes in female animals. Behavioral outcomes of control animals shown in pink, HIH-exposed animals shown in green. Points represent individual data, and solid lines represent the predicted values from the linear model. Dashed lines indicate the 95% confidence intervals for the predictions. The model included an interaction between GI and HIH exposure, with brain volume and pathology as covariates. In control females, higher motor strip GI was associated with more time in open water (*p* = 0.001; (**A**)), larger right front paw area standard deviation (*p* = 0.008; (**B**)), and decreased frequency at the center of the open field (*p* = 0.003; (**C**)). Somatosensory GI was linked to reduced total distance moved (*p* = 0.0004; (**D**)), lower exploration index (*p* = 0.01; (**E**)), and larger right front paw area (*p* = 0.009; (**F**)). These relationships were lost with HIH exposure due to significant GI and HIH interactions.

After adjusting for HIH exposure, brain volume, pathology score, and the interaction between GI and HIH exposure, the main effect for motor strip GI in females was significantly associated with time spent in open water (*p* = 0.001) in the swim test, with higher GI associated with less time spent in open water (Figure 7A). Motor strip GI was also significantly associated with larger right front paw print area standard deviation (representing increased variability and less coordinated movement, *p* = 0.008; Figure 7B) in the CatWalk and decreased frequency at the center of the open field (*p* = 0.003; Figure 7C). The main effect of somatosensory strip GI was significantly decreased total distance moved (*p* = 0.0004), decreased exploration index (*p* = 0.01) in the open-water swim test, and larger right front paw print area in the CatWalk (*p* = 0.009) in control females (Figure 7D–F). These relationships were all lost in females with HIH exposure, as the interaction between HIH exposure and GI was significant. As part of the sensitivity analysis restricting to GI range of control animals, these results remained unchanged (Supplemental Figure S7).

**Figure 8:**
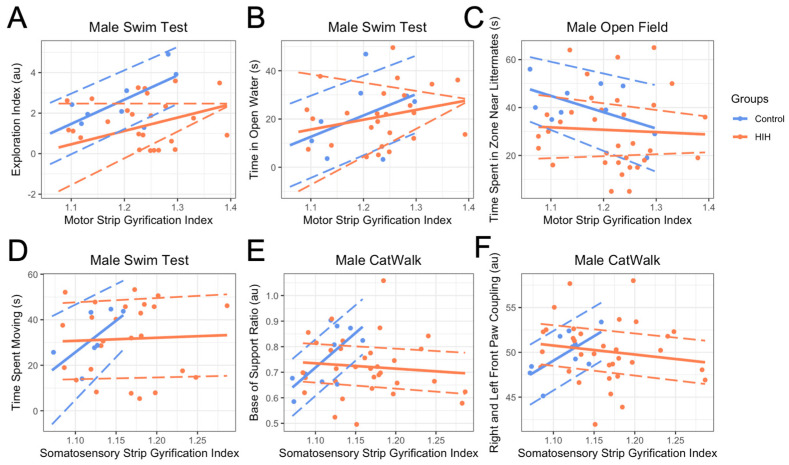
Relationship between gyrification index (GI) and behavioral outcomes in male animals. Behavioral outcomes of control animals shown in blue, HIH-exposed animals shown in orange. Points represent individual data, and solid lines represent the predicted values from the linear model. Dashed lines indicate the 95% confidence intervals for the predictions. The model includes the interaction between GI and HIH exposure, with brain volume and pathology as additional covariates. After adjusting for covariates, motor strip GI was associated with greater exploration (*p* = 0.02; (**A**)), more time in open water (*p* = 0.01; (**B**)), and less time near littermates (*p* = 0.003; (**C**)). Somatosensory GI was linked to increased swim movement (*p* = 0.03; (**D**)), wider base of support (*p* = 0.0001; (**E**)), and greater front paw coupling (*p* = 0.01; (**F**)). These associations were reduced or lost with HIH exposure, as shown by significant GI and HIH interactions.

In males, after adjusting for HIH exposure, brain volume, pathology score, and the interaction between GI and HIH exposure, the main effect of motor strip GI was significantly increased exploration index (*p* = 0.007; Figure 8A) and increased time in open water (*p* = 0.01; Figure 8B) in the swim test, and decreased time spent in the zone near littermates in the open field (*p* = 0.003; Figure 8C) in controls. After adjusting for the same covariates, somatosensory GI was significantly positively associated with time spent moving in the swim test (*p* = 0.03; Figure 8D), increased base of support (*p* = 0.0001; Figure 8E), and increased front paw coupling on the CatWalk (*p* = 0.01; Figure 8F).

Although not statistically significant, associations between GI and behavior appeared diminished in HIH-exposed males: motor GI with open-water time (interaction *p* = 0.23) and somatosensory GI with swim movement (*p* = 0.15; Figure 8B–D). Significant GI and HIH interactions indicate disrupted relationships for motor GI with exploration (*p* = 0.04; Figure 8A) and proximity to littermates (*p* = 0.09), and somatosensory GI with base of support (*p* = 0.0002; Figure 8E) and front paw coupling (*p* = 0.01; Figure 8F), suggesting HIH decouples GI from behavioral outcomes. Findings were consistent in sensitivity analyses restricting GI to control ranges (Supplemental Figure S8). The sex-specific GI and association with reflex and behavioral outcomes are summarized in Supplemental Figure S9.

We then investigated if unilateral GI better predicted the corresponding contralateral behavioral outcomes compared to GI averaged across both hemispheres, using the same covariate adjustments. In control females, left GI significantly predicted right front paw print area on the CatWalk (*p* = 0.001) while there was no association with right GI (*p* = 0.27; Supplemental Figure S10A,B). In control males, left GI trended towards predicting right front initial dual stance in the CatWalk (*p* = 0.051) similar to right GI (*p* = 0.06; Supplemental Figure S10C,D). Unilateral associations were not consistently observed across most CatWalk behavioral measures.

### 3.6. Effect of Treatment on GI Metrics and on Relationship Between GI and Behavior

Treatment with Epo, TH, or uridine did not meaningfully alter GI in males or females compared to vehicle (Supplemental Figure S11). After adjusting for pathology and brain volume, there were no differences in average or peak GI across treatments in females (Supplemental Figure S12A,B). In males, control animals had significantly lower average GI (*p* = 0.03) and similar peak GI (*p* = 0.10) compared to saline vehicle, but treatment with Epo, TH, or uridine was not associated with differences in average or peak GI (Supplemental Figure S12C,D). For some outcomes, there was evidence that treatment with Epo or TH made the relationship between GI and behavior look more similar to controls than vehicle animals in females (Supplemental Figure S13) and males (Supplemental Figure S14). For example, the interaction between motor strip GI and Epo was significant (*p* = 0.03) in predicting the time spent in open water in females, suggesting that Epo makes the slope of the line between time spent in open water and motor strip GI look more similar to controls than vehicle (Supplemental Figure S13A). A similar relationship was seen with TH in the relationship between time spent moving in the swim test and somatosensory GI in males (interaction *p* = 0.04; Supplemental Figure S14D). However, this pattern was not consistently observed across multiple related behavioral tests. For example, treatment did not significantly interact with somatosensory strip GI to predict the exploration index in females (Supplemental Figure S13E), nor with motor strip GI in males (Supplemental Figure S14A).

## 4. Discussion

Gyrification, the folding of the cerebral cortex to create a larger cortical surface area, is critical for brain development and higher cortical function, yet its relationship to early brain injury and behavior remains unknown. In this ferret model of inflammation-sensitized HI, we measured GI to assess how brain injury affects cortical folding patterns and their association with reflex and behavioral outcomes. We found that the GI of female control animals tended to be higher than male control animals throughout the cortex. Female control and HIH-exposed animals also had similar trends in GI throughout the cortex. Male control animals had a lower GI compared to females, and male HIH-exposed animals had a GI that was similar to the female animals.

The GI is a ratio of the hemispheric circumference to a trace including the curvature of the gyri and sulci; due to the nature of a ratio, the GI creates a quantitative measure of gyrification that captures more than just the differences in brain volume evident in our injury model. Furthermore, average GI and brain volume had differing relationships in control and HIH-exposed animals, supporting GI as a unique measure of brain morphology. GI could provide an additional outcome measure for clinicians, but further research is needed to decipher the differing association of GI with varying injury level in humans.

Prematurity is known to lead to decreased gyrification, reduced cortical surface area, and increased curvature of the brain [25]. Interestingly, our study observed that animals exposed to HIH exhibited increased GI compared to controls, especially in males. This probably reflects the loss of cortical tissue or white matter leading to deeper sulci rather than an increase in cortical complexity, highlighting some inherent limitations in translating findings directly to human anatomy and (patho)physiology. Cortical folding progresses with age in developing infants, leading to increased cerebral folds, enhanced gyrification, and, consequently, higher GI. In our ferret model, GI increases with escalating brain pathology until severe injury occurs, at which point GI decreases again. This eventual decrease in GI may also primarily be driven by severe tissue loss in the animals with the greatest injury. Thus, while the ferret model is valuable for studying hypoxic–ischemic brain injury, and GI may be a useful outcome for determining the success of therapies in the model, the interpretation of GI appears to differ between humans born preterm and ferrets exposed to HIH.

Previous studies have evaluated the effect of prematurity and gyrification on cognitive outcomes in humans. A study by Hedderich et al. in 2019 found that gyrification was increased in the fronto-temporo-parietal primary and associative cortices of very premature-born adults, and they found that lower gestational age was associated with increased gyrification [26]. Overall, Hedderich et al. found that gyrification, the way in which the brain’s cortical areas develop, reflected the impact of premature birth on adult intelligence quotient (IQ), and that altered gyrification was associated with lower IQ [26]. Several studies have found an association between gyrification and neurodevelopment in preterm infants [16,26,27,28]. GI has also been studied in the context of the social determinants of health; one study found that GI was significantly correlated with maternal social disadvantage, finding that higher GI was correlated with higher disadvantage factors [29]. A study of term infants with hypoxic–ischemic brain injury found that brain MRI findings could predict motor outcomes and mortality, supporting the use of brain imaging to better understand the extent of injury and implications for motor development [30]. Overall, these human data suggest that the direction of GI change (up or down) is less important than its deviation from the normative trajectory—paralleling our conclusion that the increased GI seen in HIH-exposed ferrets likely reflects maladaptive, not compensatory, cortical remodeling. Additional studies are needed to further evaluate the use of early brain imaging with long-term outcomes.

Previous research in ferrets has investigated the sexually dimorphic nature of gyrification, with males having increased GI compared to females starting on P21 and onward [15]. In humans, this trend is similar, with males having higher GIs compared to females at 2 years of age [31]. This is the opposite of what we found in our control animals at P42, which may be due to differences in ferret strain or shipping and housing conditions. As ferrets are not born in our facility, there may have been clinical risk factors at birth that resulted in these differences, similar to how lower birth weights and multiple pregnancies negatively affect cortical folding in humans [32]. It is important to note that control animals underwent minimal experimental manipulation, limited to periodic weight assessments [7]. Control animals were also equally balanced and randomized across all litters used in the study.

Sex differences relating to gyrification have also been implicated in autism spectrum disorder. Males with autism had decreased gyrification of the ventromedial/orbitofrontal prefrontal cortex [33]. Cortical thickness and gyrification are distinct and likely governed by different processes. One study found that while gyrification linearly decreased with age, cortical thickness had a more complex, nonlinear trajectory including significant sex differences [34]. Male premature newborns were also particularly vulnerable to white matter injury while females were more vulnerable to gray matter injury [35]. As white matter is particularly vulnerable to sustained or intermittent hypoxic–ischemic brain injury, males may have less resilience to injury and see greater altered GI as a result [36].

While GI metrics were not associated with reflex tests in females, motor and somatosensory strip GIs were associated with reflex development in male control animals. Furthermore, motor and somatosensory strip GI metrics were significantly associated with late behavioral test metrics in control females. Higher GI was associated with decreased time spent in open water and decreased exploration in the swim test. In ferrets, these behaviors indicate increased anxiety-like behavior characterized by thigmotaxis, as the animal stays close to the edges and does not venture into open spaces. The fact that most CatWalk outcomes related to GI involved the right forepaw was particularly interesting. The LCA is permanently ligated while the RCA is only temporarily ligated, resulting in decreased blood flow and pronounced morphological alterations of the left hemisphere, which controls the motor ability of the right paws. We found some evidence that unilateral GI predicted contralateral CatWalk outcomes, particularly involving the right forepaw. However, this relationship was not entirely consistent across hemispheric GIs and CatWalk metrics. In control males, somatosensory and motor strip GIs were also associated with several behavioral results, with higher GI being associated with decreased anxiety-like behaviors: increased exploration, time in open water, and decreased time near littermates. Across both sexes, most of the relationship between GI metrics and behavioral outcomes were lost with HIH exposure.

This sex-specific difference in the relationship between GI and anxiety-like behaviors suggests that cortical folding may influence behavior through distinct mechanisms in males and females. Since the GI of control males differs from control females, some of these associations are likely influenced by sex-specific developmental trajectories of the cortex. One possible explanation is that increased motor GI in males reflects enhanced connectivity or compensatory reorganization that supports exploratory behavior, whereas in females, increased somatosensory GI may be linked to heightened sensory processing and anxiety-like responses. Additionally, sex differences in stress reactivity and environmental sensitivity may contribute to these divergent patterns, as previous studies in ferrets and humans have shown that males and females exhibit different coping strategies in response to stressors [37,38].

Somatosensory and motor GIs correlated with behavioral outcomes in our study, but these associations were also predominately lost with HIH exposure. Existing research highlights that the suprasylvian sulcus represents the third cortical somatosensory area in ferrets, which contains a somatotopically organized representation of the ferret’s body surface as well as other higher-order somatosensory integration [39]. Furthermore, studies have shown that this sulcus is involved in the visual area and likely plays a role in motion processing, with additional evidence suggesting that it integrates complex motion processing signals across broader spatial regions [40,41,42]. In HIH-exposed animals, this sulcus deepened, extending from the midbrain posteriorly. Such changes in the suprasylvian sulcus due to HIH exposure could adversely affect visual and motion processing capabilities, as evidenced through behavioral testing, and may have contributed to our findings.

Treatment with Epo, TH, or uridine did not meaningfully alter GI across the brain or the association between GI and behavioral tests. Epo is a cytokine that stimulates erythropoiesis but also is a growth factor and neuroprotectant in the central nervous system [43]. As previously published, Epo improved cortical neuropathology and motor outcomes in this cohort of animals [7]. Therefore, we expected that it might likewise restore the typical coupling between cortical folding and behavior. We hypothesized that a hallmark of effective neuroprotective therapy would be normalization of the association between GI and a specified behavioral outcome in a sex-dependent manner, aligning more closely with the pattern observed in control animals than vehicle animals. This would be indicated by the slopes of the linear models for Epo and TH converging towards those of the control group. Although some trends supported this hypothesis, treatments did not consistently make the relationship between GI and behavioral outcomes more similar to that of control animals. While Epo has been shown to reduce demyelination after HI, treatment with Epo and TH did not show additive effects in a sheep model of ischemia, or in term newborns with HIE, suggesting overlapping anti-inflammatory and anti-apoptotic properties [44,45]. Our findings also support that these therapies do not meaningfully alter gyrification. While TH remains the standard of care for newborns with HI, additional therapies are needed to enhance neuroprotection and potentially help repair gyrification abnormalities. There were no differences in GI metrics when comparing uridine to vehicle; the lack of effect on gyrification could be due to irreversibly altered brain morphology from HI as well as mortality and severe injury seen in animals treated with uridine.

### Strengths and Limitations

Our study had several strengths, including analyzing ex vivo MRI data from over 90 animals. The ferret provides a unique model that can examine long-term effects of injury on gyrification while also displaying a gray-to-white matter ratio more similar to humans than rodent models, and lower costs than larger animals such as non-human primates [8,9,11,12,46]. Ferrets are born lissencephalic and develop gyrification postnatally [11,12,18,47]. Our model used P17 animals exposed to HIH to create a late-preterm model of HI. Importantly, we accounted for the nonlinear relationship between brain volume and GI by adjusting for brain volume as a quadratic term in all analyses, strengthening the validity of our findings. However, the results from this study may not be generalizable to extremely preterm models, especially considering the complex and often nonlinear trends seen with white matter maturation [48]. Another limitation of the study is that animal numbers were determined based on the primary outcome study [7]. As such, it is possible that we were underpowered to detect certain sex-dependent relationships or responses to treatment after accounting for multiple variables and interactions in our models. Future studies will need to ensure reproducibility, especially in other gyrencephalic animal models. While GI provides a global measure of cortical folding, complementary imaging metrics such as sulcal depth and local curvature could help disentangle whether increased GI reflects maladaptive folding (e.g., shallow, irregular sulci or aberrant convexity) versus compensatory folding patterns. These regional metrics would allow us to better distinguish pathological over-folding from adaptive plasticity, helping to clarify whether the observed increases in GI represent injury-driven distortion or recovery processes.

## 5. Conclusions

In our ferret model of HI, GI is notably altered in male animals only, driven by male HIH-exposed animals displaying increased gyrification indices similar to female animals. Juvenile ferrets display sex-specific relationships between regional GI and behavioral test performance; higher GI may be associated with faster reflex development in males and increased anxiety-like behavior in females. Several GI metrics in the somatosensory and motor strips of control animals were associated with reflex and behavioral outcomes, indicating GI may be an important outcome measure for use in future neuroprotection studies in the ferret, including when examining outcomes by sex. Normalization of the relationship between GI and behavior may be an important outcome measure in future neuroprotection studies, but it did not appear to be consistently affected by either TH or Epo.

## Data Availability

The data described in the manuscript and/or analyzed during the current study will be made available upon reasonable request from the corresponding author.

## Supplementary Materials

The following supporting information can be downloaded at: https://www.mdpi.com/article/10.3390/life15091428/s1. Figure S1: Smoothed quantile regression showing median gyrification index (GI) spanning the brain comparing left and right hemispheres; Figure S2: Association between average gyrification index (GI) and brain volume in control and HIH-exposed animals; Figure S3. Quadratic regression showing association between gyrification index (GI) and pathology score in female and male animals; Figure S4: Quadratic regressions showing the association of pathology score and regional gyrification index (GI), brain volume, and regional GI in control and HIH-exposed animals; Figure S5: Graphical networks of somatosensory and motor gyrification index (GI); Figure S6: Relationship between motor strip gyrification index (GI) and somatosensory strip GI and with total area under the curve (AUC) of reflex testing in females and males, only including GIs within the range of control animals; Figure S7: Relationship between gyrification index and behavioral outcomes in female animals, only including GIs within the range of female control animals; Figure S8: Relationship between gyrification index and behavioral outcomes in male animals, only including GIs within the range of male control animals; Figure S9: Overview of sex-specific differences in gyrification index (GI) and their associations with reflex and behavioral outcomes; Figure S10: Relationship between unilateral gyrification index (GI) and behavioral outcomes in females (top row) and males (bottom row); Figure S11: Smoothed quantile regression showing median gyrification index (GI) spanning the brain comparing control, erythropoietin (Epo), therapeutic hypothermia (TH), and saline vehicle in females and males; Figure S12: Average and peak gyrification index (GI) across treatment groups split by sex; Figure S13: Relationship between gyrification index and behavioral outcomes in female animals split by treatment; Figure S14: Relationship between gyrification index and behavioral outcomes in male animals split by treatment.

## Abbreviations

The following abbreviations are used in this manuscript:

AIC	Akaike information criterion
AUC	Area under the curve
Epo	Erythropoietin
GI	Gyrification index
H&E	Hematoxylin and eosin
HI	Hypoxia–ischemia
HIH	Hypoxia–ischemia with hyperoxia
IACUC	Institutional Animal Care and Use Committee
IQ	Intelligence quotient
IQR	Interquartile range
LCA	Left carotid artery
LPS	Lipopolysaccharide
MRI	Magnetic resonance imaging
P	Postnatal day
p	Probability
PBS	Phosphate-buffered saline
RCA	Right carotid artery
TH	Therapeutic hypothermia
