# Supplementary material for: Ferreting Out the Effects of Neonatal Hypoxia–Ischemia and Sex on Ferret Cortical Gyrification
Source: Life (Basel). 2025 Sep 11;15(9):1428. doi: 10.3390/life15091428 (PMC12471414; doi:10.3390/life15091428)
Supplement: Supplementary file 1 [file life-15-01428-s001.zip › life-3837808-supplementary.pdf]

Supplemental Methods

Statistical Analysis

Smoothed quantile regressions were used to show the median gyrification index (GI) spanning the brain comparing left and right hemispheres. Mann Whitney U-tests were used to test for differences in hemispheric average and peak GIs within the same sex and hypoxia-ischemia with hyperoxia (HIH) exposure. Graphical networks of the top 15 variables associated with somatosensory or motor strip GI for control and HIH-exposed animals used Pearson correlation coefficients. Linear regression with robust standard error adjusting for pathology and brain volume was used to assess differences in average and peak GI across treatments, stratified by sex. The relationships between GI and behavioral outcomes included adjustment for injury (HIH-exposed versus control), brain volume, pathology score, the interaction between GI and HIH exposure, and were stratified by sex. Linear regressions with robust standard errors were used, and scatter plots show the predicted marginal linear estimate with 95% confidence intervals. This method was also used to assess the relationship between unilateral GI on unilateral behavioral outcomes.

Supplemental Figures

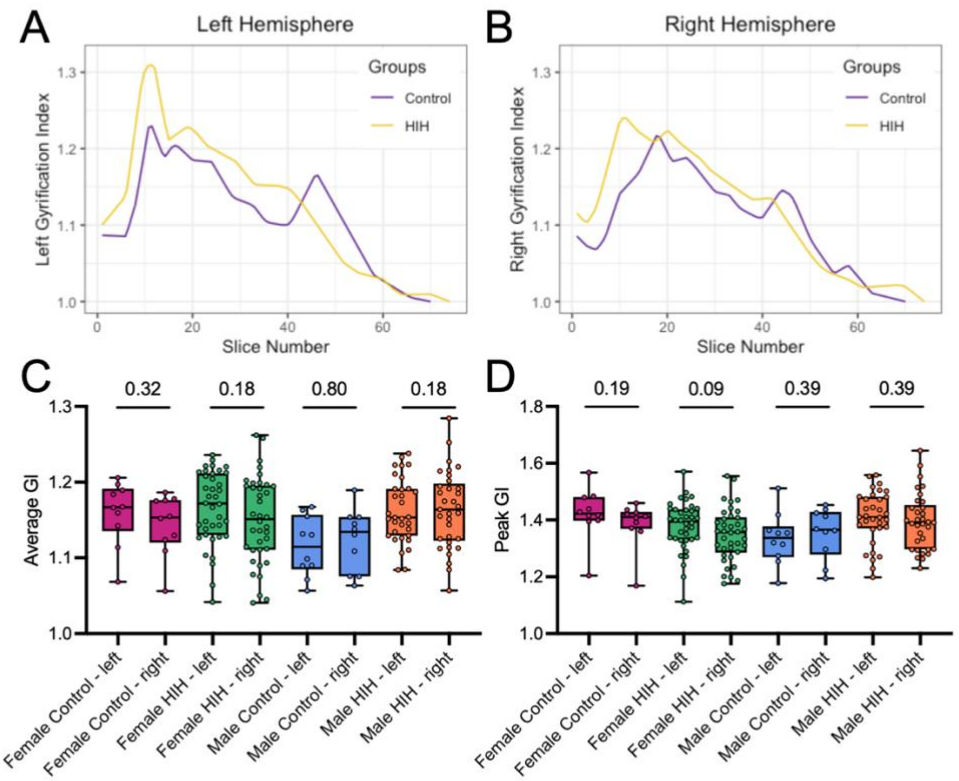

Supplemental Figure 1. Smoothed quantile regression showing median gyrification index (GI) spanning the brain comparing left (A) and right (B) hemispheres in control (purple) and hypoxia-ischemia with hyperoxia (HIH) exposed (gold) animals. Box plots display the median, interquartile range, and overall range show average GI (C) and peak gyrification index (D) grouped by sex, HIH exposure, and hemisphere. No significant hemispheric differences were observed. Mann Whitney U tests were used.

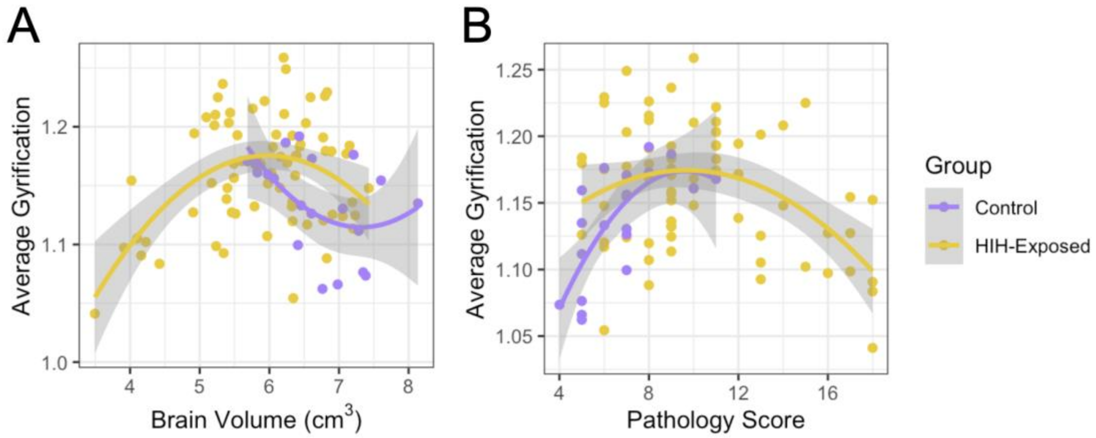

Supplemental Figure 2. Association between average gyrification index (GI) and brain volume (A) and pathology score (B) in control (purple) and hypoxia-ischemia with hyperoxia (HIH) exposed (gold) animals. In control animals, increasing average GI trended towards decreased brain volume (p=0.06) and increased pathology (p=0.002) using quadratic regression with robust standard error. In HIH-exposed animals, average GI was significantly associated with brain volume (p=0.0001) and pathology score (p=0.0007) using quadratic regression with 95% confidence interval shaded.

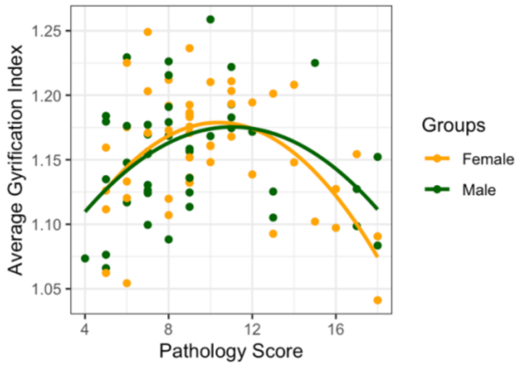

Supplemental Figure 3. Quadratic regression showing association between gyrification index (GI) and pathology score in female (orange) and male (green) animals. Female and male animals exhibited a similar quadratic association between GI and pathology score.

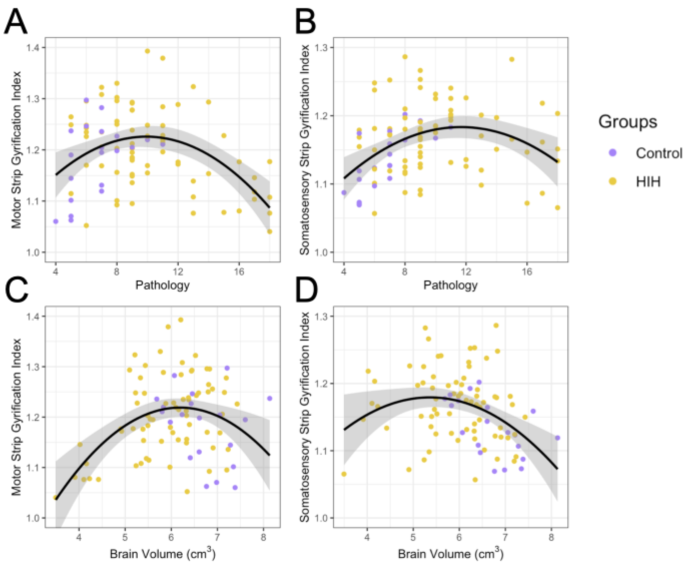

Supplemental Figure 4. Quadratic regressions showing the association of pathology score with motor strip gyrification index (GI) (A) and somatosensory strip GI (B), and brain volume with motor strip GI (C) and somatosensory strip GI (D) in control (purple) and HIH-exposed animals (yellow). 95% confidence interval shaded. Motor and somatosensory GI increased with increasing pathology and brain volume but decreased once injury reached a severe level.

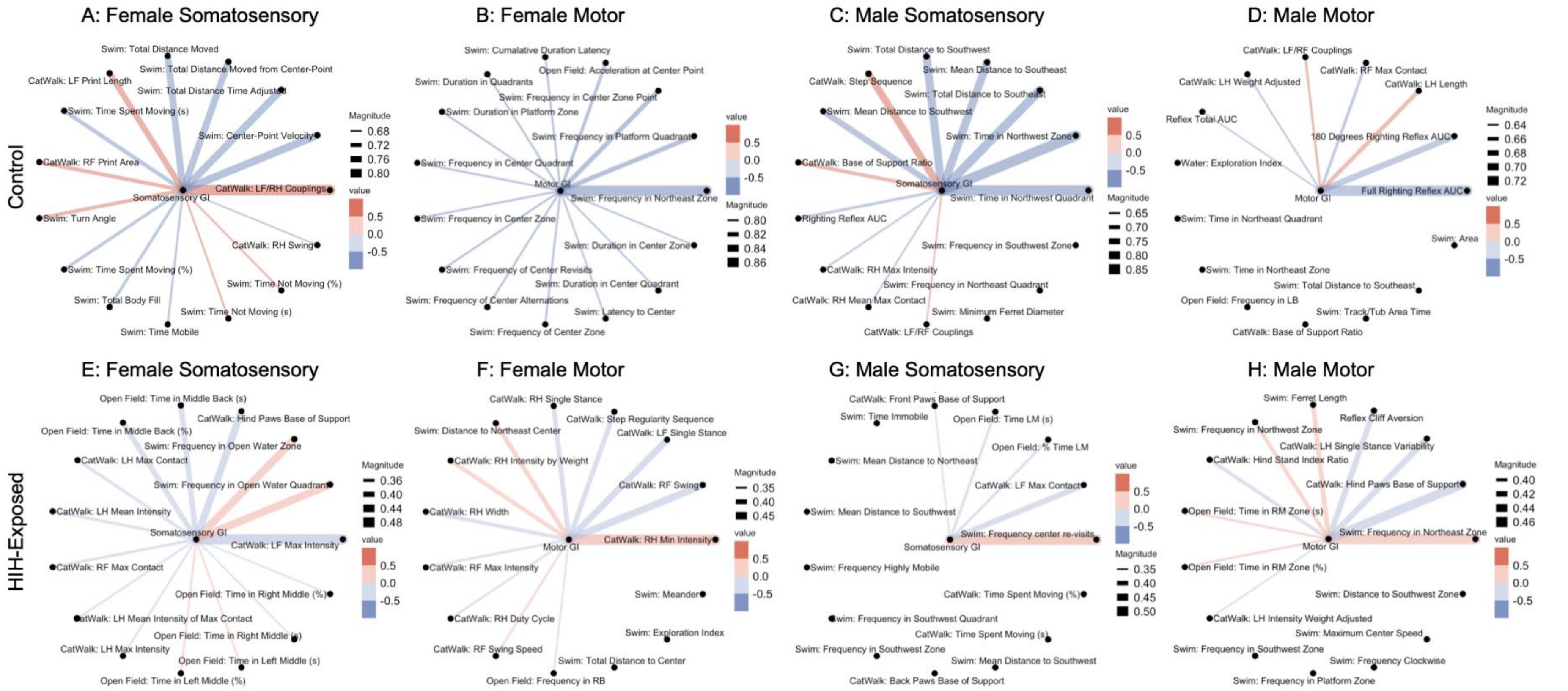

Supplemental Figure 5. Graphical networks of somatosensory and motor gyrification index (GI). The top 15 variables associated with somatosensory or motor strip GI for control animals (top row) and HIH-exposed animals (bottom row) shown. Non-significant correlations ($p \geq 0.05$) are whited out to emphasize only statistically significant relationships. Lines connecting two points represent statistically significant relationships. Red lines denote significant positive associations between morphological parameters and conditioning, while blue lines indicate significant negative associations. The thickness of each line reflects the strength of the relationship. Overall, there were more significant associations between GI and behavioral outcomes in control animals compared to HIH-exposed animals, except for the motor strip GI in males.

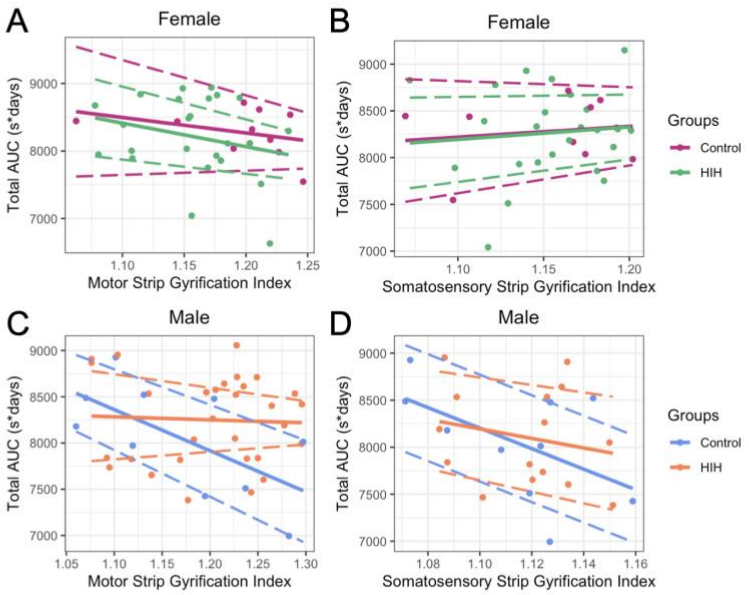

Supplemental Figure 6. Relationship between motor strip gyrification index (GI) (A, C) and somatosensory strip GI (B, D) with total area under the curve (AUC) of reflex testing in females (top row) and males (bottom row), respectively, only including GIs within the range of control animals. Points represent individual data, and solid lines represent the predicted values from the linear model. Dashed lines indicate the 95% confidence intervals for the predictions. Only animals with GIs within the range of control animals were included. The model includes the interaction between GI and HIH exposure, with brain volume and pathology as additional covariates. After adjusting for HIH exposure, brain volume, pathology score, and the interaction between GI and HIH exposure, GI was not associated with reflex testing AUC in female animals. Somatosensory strip ($p=0.045$) GI was only significantly associated with total reflex testing AUC in male control animals, with higher GI associated with faster reflex testing times.

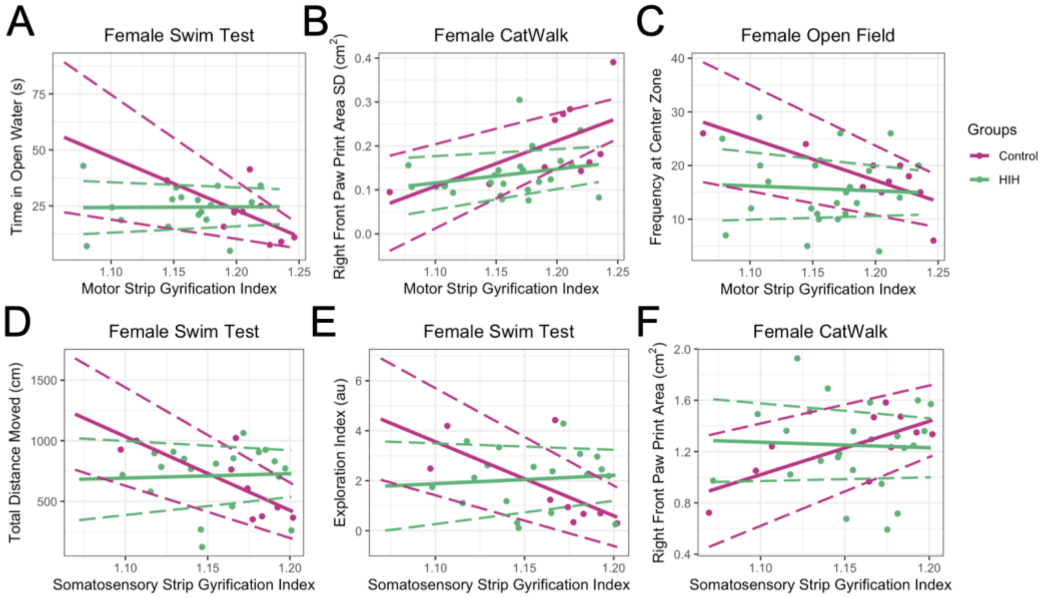

Supplemental Figure 7. Relationship between gyrification index (GI) and behavioral outcomes in female animals, only including GIs within the range of female control animals. Behavioral outcomes of control animals shown in pink, HIH-exposed animals shown in green. Points represent individual data, and solid lines represent the predicted values from the linear model. Dashed lines indicate the 95% confidence intervals for the predictions. Only animals with GIs within the range of female control animals were included. The model includes the interaction between GI and HIH exposure, with brain volume and pathology as additional covariates. In control females, higher motor strip GI was associated with more time in open water, larger right front paw area standard deviation, and greater acceleration at the center of the open field. Somatosensory GI was linked to reduced total distance moved, lower exploration index, and larger right front paw area. These relationships were reduced or lost with HIH exposure, as shown by significant GI and HIH interactions.

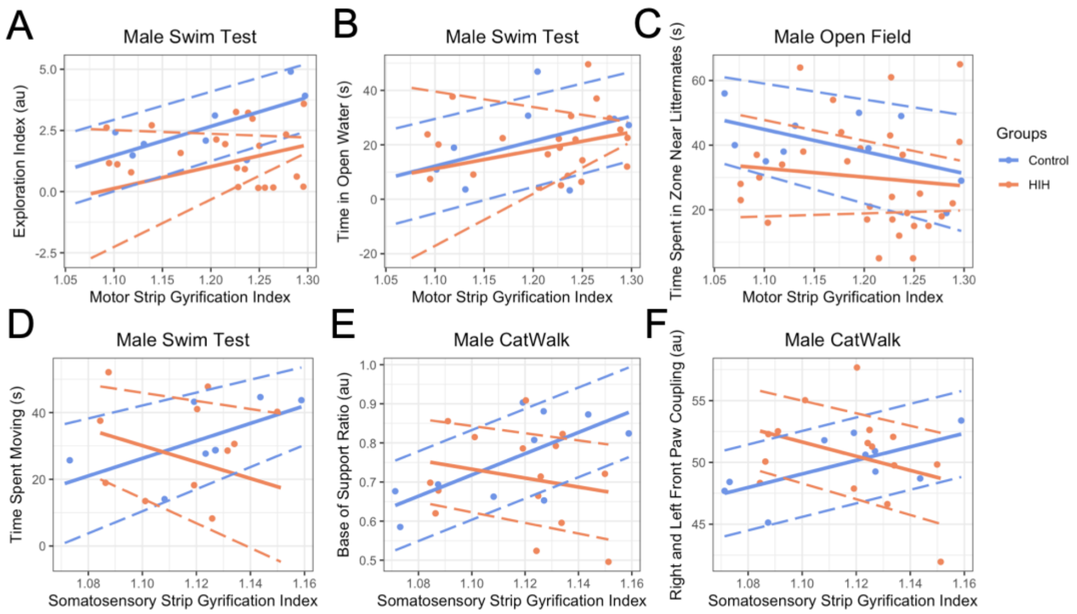

Supplemental Figure 8. Relationship between gyrification index (GI) and behavioral outcomes in male animals, only including GIs within the range of male control animals. Behavioral outcomes of control animals shown in blue, HIH-exposed animals shown in orange. Points represent individual data, and solid lines represent the predicted values from the linear model. Dashed lines indicate the 95% confidence intervals for the predictions. Only animals with GIs within the range of male control animals were included. The model includes the interaction between GI and HIH exposure, with brain volume and pathology as additional covariates. After adjusting for covariates, motor strip GI was associated with greater exploration, more time in open water, and less time near littermates. Somatosensory GI was linked to increased swim movement, wider base of support, and greater front paw coupling. These associations were reduced or lost with HIH exposure, as shown by significant GI and HIH interactions.

	Faster Reflex Development	Anxiety Behavioral Outcomes
**Male Control**	↑ Motor<br>↑ Somatosensory	↓ Motor<br>↓ Somatosensory
**Male HIH**	↑ Motor	Varied*
**Female Control**	No Relationship	↑ Motor<br>↑ Somatosensory
**Female HIH**	No Relationship	No Relationship

Supplemental Figure 9. Overview of sex-specific differences in gyrification index (GI) and their associations with reflex and behavioral outcomes. Upward arrows indicate increased GI, and downward arrows indicate decreased GI. *Some behavioral outcomes demonstrated significant interactions between GI and HIH, while others did not.

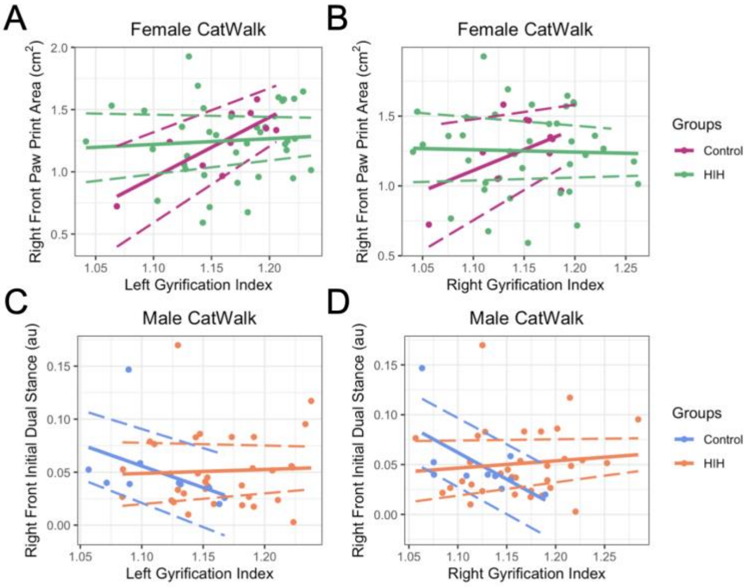

Supplemental Figure 10. Relationship between unilateral gyrification index (GI) and behavioral outcomes in females (top row) and males (bottom row). Points represent individual data, and solid lines represent the predicted values from the linear model. Dashed lines indicate the 95% confidence intervals for the predictions. The model includes the interaction between GI and HIH exposure, with brain volume and pathology as additional covariates. In control females, left GI significantly predicted right front paw print area on the CatWalk (p=0.001) while there was no association with right GI (p=0.26). In control males, left GI trended towards predicting right front initial dual stance in the CatWalk (p=0.052) similar to right GI (p=0.06).

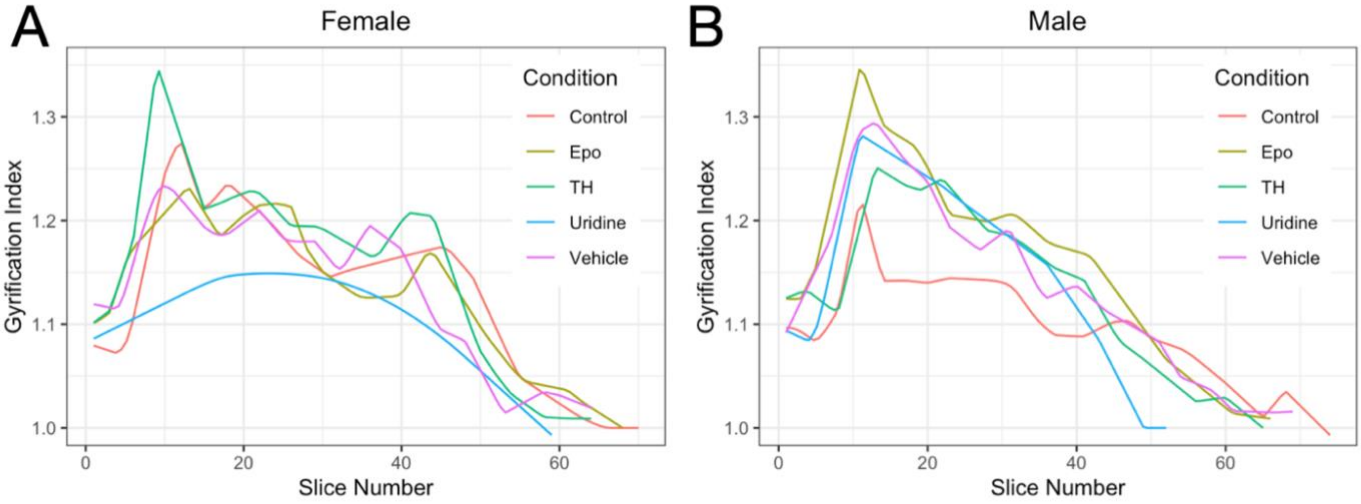

Supplemental Figure 11. Smoothed quantile regression showing median gyrification index (GI) spanning the brain comparing control, erythropoietin (Epo), therapeutic hypothermia (TH), and saline vehicle in females (A) and males (B). Treatment did not meaningfully alter GI across the cortex.

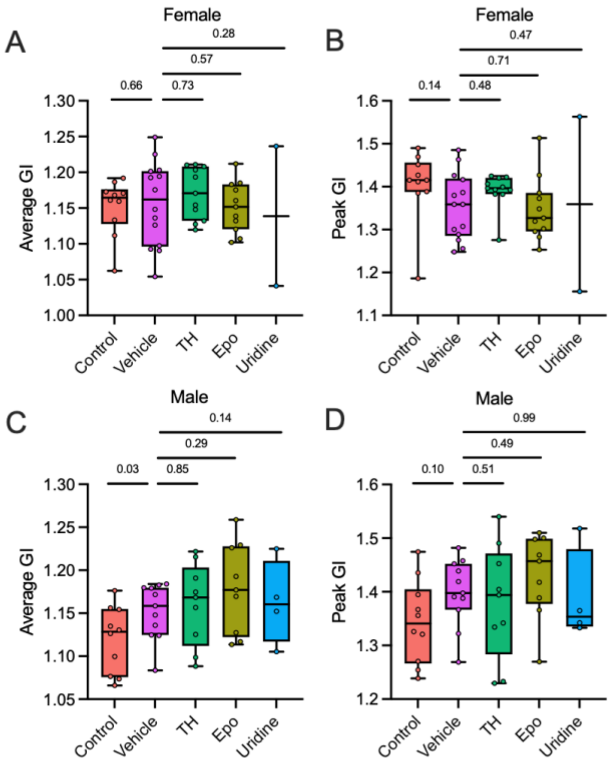

Supplemental Figure 12. Average and peak gyrification index (GI) across treatment groups split by sex. Linear regression with robust standard error adjusting for pathology and brain volume used for both sexes. Treatment was not associated with average (A) or peak (B) GI in females. In males, control animals had significantly lower average GI (C) and similar peak GI (D). Treatment with therapeutic hypothermia (TH), erythropoietin (Epo), or Uridine was not associated with average or peak GI. Data are presented as a box plot, showing the median, interquartile range, and overall range.

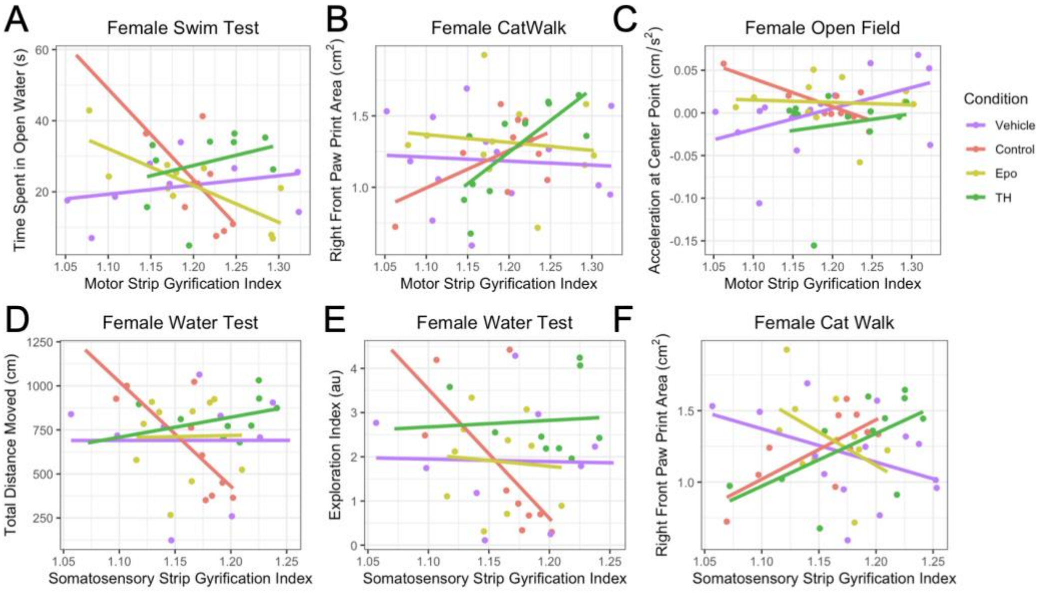

Supplemental Figure 13. Relationship between gyrification index and behavioral outcomes in female animals split by treatment. Points represent individual data, and solid lines represent the predicted values from the linear model. The model includes the interaction between GI and treatment, with brain volume and pathology as additional covariates. The interaction between motor strip GI and erythropoietin (Epo) was significant ($p = 0.03$) in predicting the time spent in open water in females, suggesting that Epo makes the slope of the line between time spent in open water and motor strip GI look more similar to controls than Vehicle (Panel A). However, this pattern was not consistently observed across multiple related behavioral tests. For example, treatment did not significantly interact with somatosensory strip GI to predict the exploration index in females (Panel E).

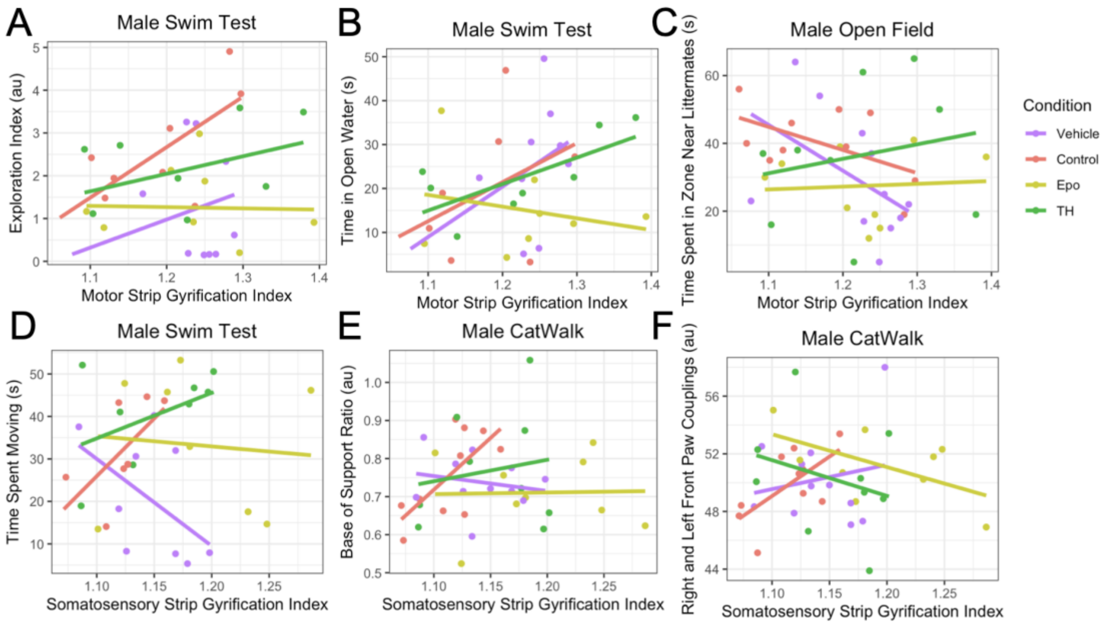

Supplemental Figure 14. Relationship between gyrification index (GI) and behavioral outcomes in male animals split by treatment. Points represent individual data, and solid lines represent the predicted values from the linear model. The model includes the interaction between GI and treatment, with brain volume and pathology as additional covariates. The interaction between somatosensory strip GI and therapeutic hypothermia (TH) was significant ($p = 0.04$) in predicting the time spent moving in open water in males, suggesting that TH makes the slope of the line between time spent moving in open water and somatosensory strip GI look more similar to controls than Vehicle (Panel D). However, this pattern was not consistently observed across multiple related behavioral tests. For example, treatment did not significantly interact with motor strip GI to predict exploration index in males (Panel A).
